# Spontaneous C-cleavage of a truncated intein as fusion tag to produce tag-free VP1 inclusion body nanoparticle vaccine against CVB3-induced viral myocarditis by the oral route

**DOI:** 10.1186/s12934-019-1115-z

**Published:** 2019-04-04

**Authors:** Xingmei Qi, Qian Lu, JingPing Hu, Sidong Xiong

**Affiliations:** The Jiangsu Key Laboratory of Infection and Immunity, Institutes of Biology and Medical Sciences, Soochow University, Suzhou, 215123 Jiangsu China

**Keywords:** Intein cleavage, Tag-free inclusion body particles, CVB3-induced viral myocarditis, Oral protein vaccine

## Abstract

**Background:**

Oral vaccine is highly desired for infectious disease which is caused by pathogens infection through the mucosal surface. The design of suitable vaccine delivery system is ongoing for the antigen protection from the harsh gastric environment and target to the Peyer’s patches to induce sufficient mucosal immune responses. Among various potential delivery systems, bacterial inclusion bodies have been widely used as delivery systems in the field of nanobiomedicine. However, a large number of heterologous complex proteins could be difficult to propagate in *E. coli* and fusion partners are often used to enhance target protein expression. As a safety concern the fusion protein need to be removed from the target protein to get tag-free protein, especially for the production of protein antigen in vaccinology. Until now, there is no report on how to remove fusion tag from inclusion body particles in vitro and in vivo. Coxsackievirus B3 (CVB3) is a leading causative agent of viral myocarditis and orally protein vaccine is high desired for CVB3-induced myocarditis. In this context, we explored a tag-free VP1 inclusion body nanoparticles production protocol though a truncated *Ssp DnaX* mini-intein spontaneous C-cleavage in vivo and also exploited the VP1 inclusion bodies as an oral protein nanoparticle vaccine to protect mice against CVB3-induced myocarditis.

**Results:**

We successfully produced the tag-free VP1 inclusion body nanoparticle antigen of CVB3 and orally administrated to mice. The results showed that the tag-free VP1 inclusion body nanoparticles as an effective antigen delivery system targeting to the Peyer’s patches had the capacity to induce mucosal immunity as well as to efficiently protect mice from CVB3 induce myocarditis without any adjuvant. Then, we proposed the use of VP1 inclusion body nanoparticles as good candidate for oral vaccine to against CVB3-induced myocarditis.

**Conclusions:**

Our tag-free inclusion body nanoparticles production procedure is easy and low cost and may have universal applicability to produce a variety of tag-free inclusion body nanoparticles for oral vaccine.

**Electronic supplementary material:**

The online version of this article (10.1186/s12934-019-1115-z) contains supplementary material, which is available to authorized users.

## Background

Traditional vaccines have been developed using inactivated or live attenuated pathogens. Recently, in the quest for biosafety, recombinant subunit vaccines based on protein are provoking wide interest. However, these vaccines have relatively low immunogenicity because of size, degradation, lack of cross-presentation, and other issues. Chief among the solutions to these problem is the need for adjuvants and/or the use of vaccine delivery systems [1–5], which may have some disadvantages including side effects and safety concerns. As one potential alternative, nanoparticles have several applications in nanobiomedicine, especially in the field of vaccinology where they can be applied as either a delivery systems to enhance antigen processing and/or as an adjuvants or immune potentiators [6–8]. A number of approved nano-sized vaccine and drug delivery systems have been approved for human use and more are in clinical or pre-clinical trials [9–14]. According to the nanomaterial composition, the vaccine associated nanoparticles could be classified in different types, including polymers, liposomes, virus-like particles (VLP), self-assembling peptides, and inorganic nanoparticles [7, 8, 12, 15–17]. Among the various nano-biomaterials, bacterial inclusion bodies (IBs) appear to be a good candidate as nano-sized protein vaccine, which are mechanically stable, non-toxic, fully biocompatible and regulatable size [18–21]. They are naturally formed by recombinant proteins in bacterial cells under overexpressing conditions. IBs is majorly composed of recombinant protein (between 70 and 95%), but it also contains different bacterial components such as chaperones, lipids, cell wall fragments as well as nucleic acids that might be further removed by convenient downstream procedures [20, 22–24]. Noteworthy, these elements have been proven to act as adjuvants or immune potentiators [25–27]. In this regard, IBs have been adapted as “all in one” nanopills [28, 29].

Bacterial is the most favorite expression system employed for the production of recombinant proteins. However, since it is a prokaryotic based system, a mount of heterologous complex proteins could be difficult to propagate [30]. For the difficult-to-express proteins, fusion partners are often used jointly to achieve efficient protein expression, as these proteins have the capacity to enhance the expression and stability of the target protein [31]. Then the tag usually needs to be removed from the final product by enzymatic or chemical cleavage at the junction between the tag and the target protein, as fusion tags can interfere in various physicochemical properties and immunogenicity of the recombinant proteins [32–35]. Usually, the recombinant proteins expressed as inclusion bodies were purified by isolation of IBs followed by solubilization and refolding, then removed the fusion tags [36–38]. However, for the purpose of expression as nanostructure materials, IBs need to be separated and purified as protein particles. Then, a wide range of protocols for obtaining of high pure and bacterial-free protein particles are available by convenient downstream procedures [20, 22, 24], but we have no option to remove the fusion tags from IBs. The intein-mediated self-cleavage system has been recently developed as a powerful tool for protein expression, purification, ligation and cyclization [39–43]. For this purpose, the in vivo self-cleavage between the target protein and the intein should be avoided during protein expression [44]. On the contrary, we explore the intein in vivo self-cleavage as a fusion tag to produce tag-free inclusion body nanoparticles in *E. coli*. In previous study, we have reported that the truncated *Ssp DnaX* mini-intein could undergo spontaneous C-cleavage without addition of its complementary N-intein. Especially, when we deleted the N-terminal 12-aa sequence of the *Ssp DnaX* mini-intein, the remaining 138-aa C-intein (Ic_138_) sequence would undergo spontaneous C-cleavage nearly completely [45]. In this study, we chose Coxsackievirus B3 (CVB3) capsid protein-1 (VP1) as a model protein to demonstrate the truncated *Ssp DnaX* mini-intein as an in vivo self-cleavage system to produce tag-free IBs.

CVB3 is an important pathogen that induces acute and chronic myocarditis which invades host via gastrointestinal mucosa. However, there is no efficient prevention vaccine available currently. Oral vaccines can induce both mucosal and systemic immune responses to offer greater efficacy against pathogens initiating the infection process at mucosal surfaces [46]. However, oral vaccine development has been hampered by antigen delivery strategies such as poor antigen uptake and rapid degradation by protease at the mucosa [47, 48]. Therefore, the development of oral vaccines is particularly important and urgently required to prevent CVB3-induced myocarditis. VP1 is an immunodominant structural protein and the efficient protein expression of VP1 in *E. coli* needs to be fused with fusion tag as IBs. In this context, we prepared and explored the tag-free VP1 IBs as protein-based nanoparticle vaccine for the production and delivery of protective antigens to mucosal surfaces to prevent CVB3-induced myocarditis by oral route.

## Results

### In vivo spontaneous C-cleavage assay

**Fig. 1:**
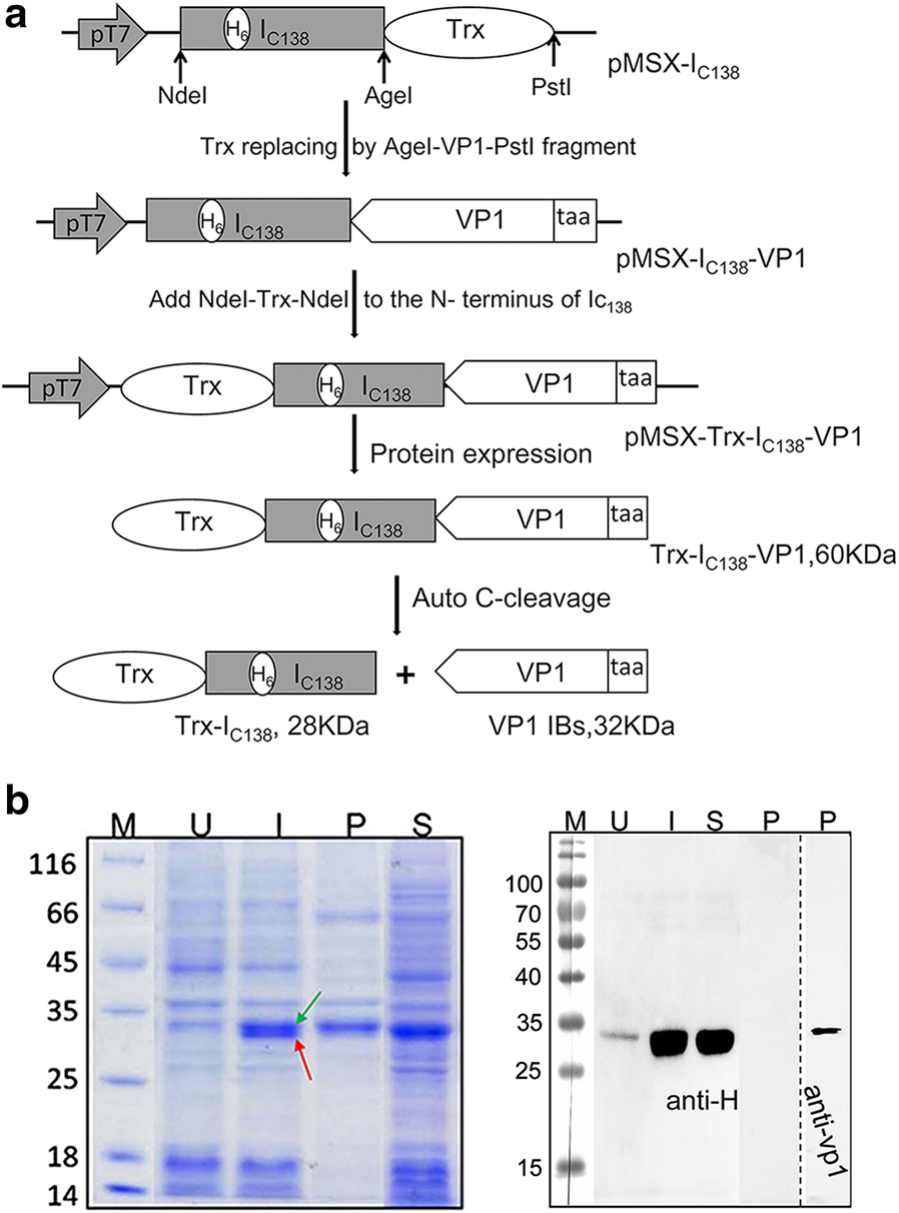
Schematic illustration of plasmid constructions and intein spontaneous C-cleavage assay in vivo. **a** Plasmid pMSX-I_C138_-VP1 was derived from previously described plasmid pMSX-I_C138_T [45] by replacing the Trx coding sequence with a VP1 sequence. Plasmid pMSX-Trx-I_C138_-VP1 was derived from plasmid pMSX-I_C138_-VP1 by adding a Trx fragment to the N terminus of I_C138_ gene sequence. The recombinant fusion protein Trx-I_C138_-VP1 consisted of Trx, Ic_138_ and VP1, with Trx being a thioredoxin protein, Ic_138_ being the 138-aa C-terminal part of the Ssp DnaX mini-intein, and VP1 being the Coxsackievirus B3 capsid protein-1. The Ic_138_ contained a 6xHis-tag (H_6_) as indicated. Spontaneous C-cleavage at the C-terminus of Ic_138_ would produce the two protein products as illustrated. **b** SDS–PAGE (left) and Western blotting (right) analysis of the C-cleavage of recombinant protein Trx-Ic_138_-VP1. Lane M: protein size markers, with their sizes shown in kDa. Lanes U and I: total cellular proteins of *E. coli* before and after IPTG-induced expression the Trx-Ic_138_-VP1 protein, respectively. Lanes P and S: the pellets and supernatants of the bacterial cell lysate after sonication, respectively. Predicted sizes of Trx-Ic_138_-VP1, Trx-Ic_138_ and VP1 are 60 kDa, 28 kDa and 32 kDa, respectively

To investigate whether the spontaneous C-cleavage reaction could occur between the Ic and VP1 in vivo, we constructed plasmid pMSX-Trx-I_C138_-VP1, which added Trx as a fusion partner to the N terminus of I_C138_ for enhancing protein expression. As effective cleavage depends on a functional intein, the intein needs to be expressed in a soluble correctly folded form. Then protein expression was performed at low temperature and low IPTG concentration (25 °C, IPTG 0.25 mM). As illustrated in Fig. 1a, intein C-cleavage would convert the precursor protein Trx-I_C138_-VP1 (60 kDa) into Trx-I_C138_ (28 kDa) and VP1 (32 kDa). As shown in Fig. 1b, compared to the un-induction protein (U), there two additional protein bands (Trx-I_C138_, red arrow and VP1, green arrow) were identified after induction (I) by their apparent sizes in SDS–PAGE. Furthermore, the induced total protein (I) was analyzed by Western blotting using anti-H antibody and no precursor protein was observed. The result indicated that the fusion protein Trx-I_C138_-VP1 was almost completely converted into cleavage products Trx-I_C138_ and VP1 through spontaneous C-cleavage in vivo. Following bacterial cell disruption, the soluble (S) and insoluble cell fractions (P) were separated by centrifugation and analyzed with SDS-PAGE and Western blotting using anti-H and anti-VP1 antibody, respectively. The cleavage product Trx-I_C138_ is in the soluble fraction, while the VP1 is in the pellets as inclusion bodies (VP1 IBs).

### Purification and characterization of tag-free VP1 IBs

**Fig. 2:**
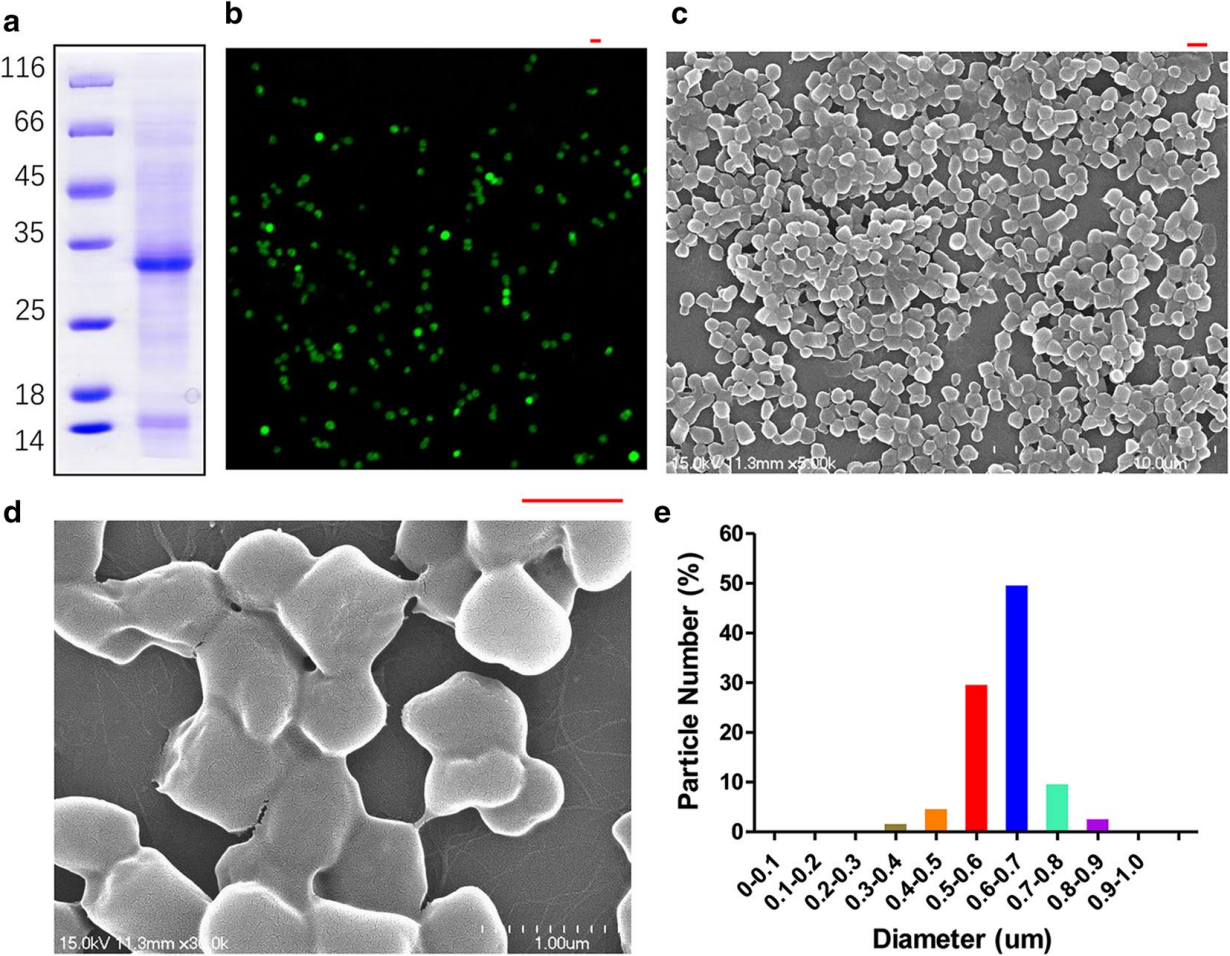
VP1 IB nanoparticles purification and characterization. **a** SDS-PAGE analysis of the purified VP1 IBs. **b** Confocal microscopy images of FITC-labeled VP1 IBs. **c**, **d** Scanning electron microscopy (SEM) images of VP1 IBs. **e** Size distribution of VP1 IBs. Scale bar is 1um

The tag-free VP1 IBs were successfully purified from the pellets as shown in Fig. 2a by SDS-PAGE analysis. For nanoparticles morphometry (size and shape) of VP1 IBs were characterized by confocal microscopy (Fig. 2b) and by SEM (Fig. 2c, d). The VP1 IBs is cylindrical with a smooth surface and the particles with a diameter is between 300 and 900 nm, peaking in the range between 500 and 700 nm (Fig. 2e).

### BMDCs uptake VP1 IBs and mediated T cell proliferation

**Fig. 3:**
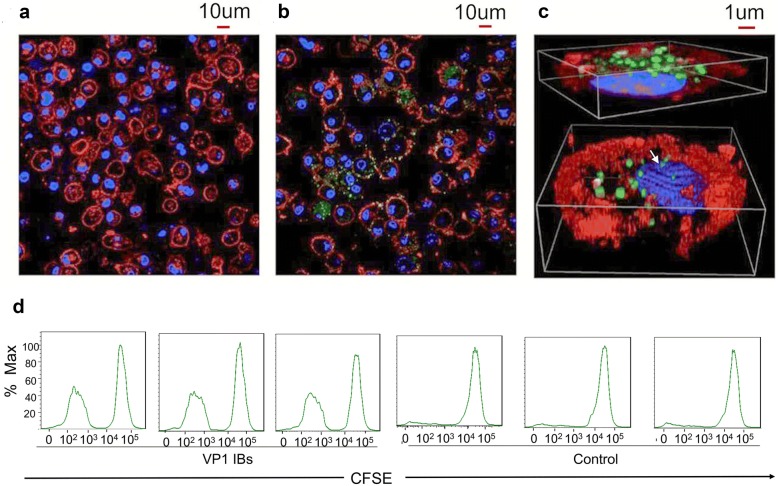
Uptake of VP1 IBs by BMDCs and T cell proliferation assay in vitro. Confocal microscopy image of BMDCs in absence (**a**) and in presence (**b**) of FITC-labeled VP1 IBs. **c** *XYZ* overlay confocal image of FITC-labeled VP1 IBs uptake by BMDCs, showing intracellular penetration of VP1 IBs. Nuclear membrane is seen in blue and cell membrane in red, VP1 IBs are seen in green. **d** Representative flow cytometry histograms of CFSE-labeled T cells show increased proliferation capacity when cultures in the presence of BMDCs loaded with the VP1 IBs, compared to control

One of the main propertied of IBs as nanoparticle protein vaccine is their ability to efficiently uptake by antigen presenting cells (APCs) and subsequently induce their maturation and cross presentation for activation of a potent immune response. For that we studied the interaction of VP1 IBs with DC cells in vitro. The results showed that BMDCs were able to uptake VP1 IBs very efficiently (Fig. 3b). At 12 h post-exposure, more than one particle per cell was usually observed. The VP1 IBs appeared intimately interacting with cell membranes. In addition, some protein particles were fully internalized by BMDC cells and the 3D images demonstrated the complete internalization of IBs with the nuclear membrane (Fig. 3c, arrow indicated). Also during the interaction with cells, the VP1 IBs maintained their usual morphology, which indicated that the stability, proteolytic resistance and functionality of the polypeptides forming IBs, in the extracellular media and also during internalization. Simultaneous, we observed a significant increase in DC-mediated T cell proliferation over 72 h in response to the VP1 IBs (Fig. 3d). These results indicated that the VP1 IB nanoparticles could be efficiently uptake by BMDCs and have the ability to induce DC-mediated T cell proliferation.

### VP1 IBs had no toxicity to cells and animals

**Fig. 4:**
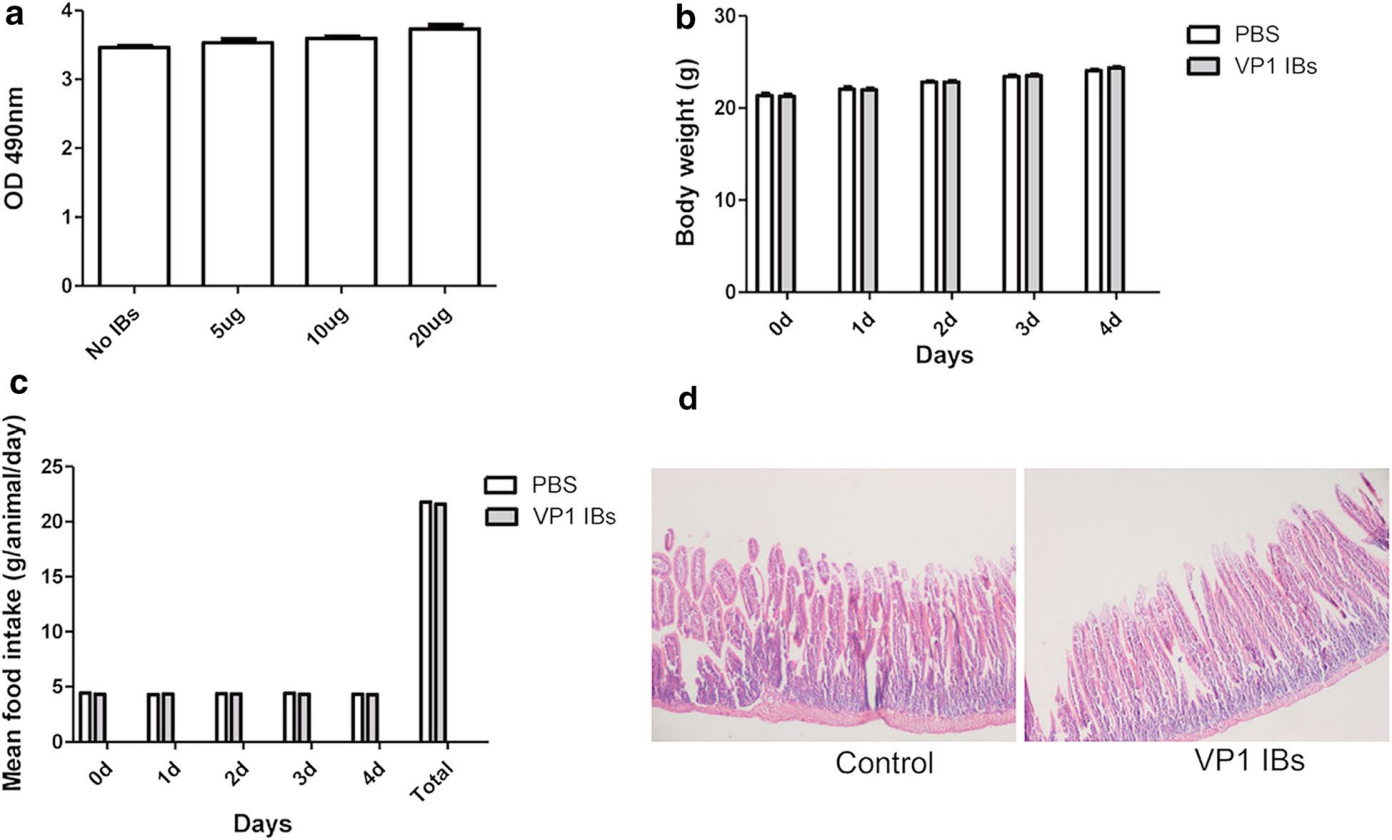
VP1 IBs cytotoxicity assay and tolerance of mice to oral VP1 IBs administration. **a** Cultured HeLa cell growth in absence and in presence of increasing amounts of VP1 IBs added to the culture media. **b** Body weight of animals during oral administration of VP1 IBs compared with control mice. **c** Food intake (per day and accumulated values) of mice during oral administration of VP1 IBs compared with control mice. **d** Histological sections of the intestine of VP1 IBs oral administered mice and control mice

For safety concerns, the toxicity of VP1 IBs were tested in cells and animals. A cytotoxicity test was performed on HeLa cells exposed to various VP1 IBs concentration. After 24 h of incubation, the different concentration of VP1 IBs did not promote any evident sign of toxicity upon their addition to the culture media, based on the qualitative comparison of cell density between controls and IB treated cell cultures (Fig. 4a). And then, we tested the VP1 IBs tolerance to oral administration to mice. In repeated administration, VP1 IBs were well tolerated by all animals showing no weight loss (Fig. 4b), normal food intake (Fig. 4c) and no histological damage in the intestine in IB-fed animals compared to control group (Fig. 4d).

### Oral immunization with VP1 IBs elicited strong CVB3-specific mucosal immunity

**Fig. 5:**
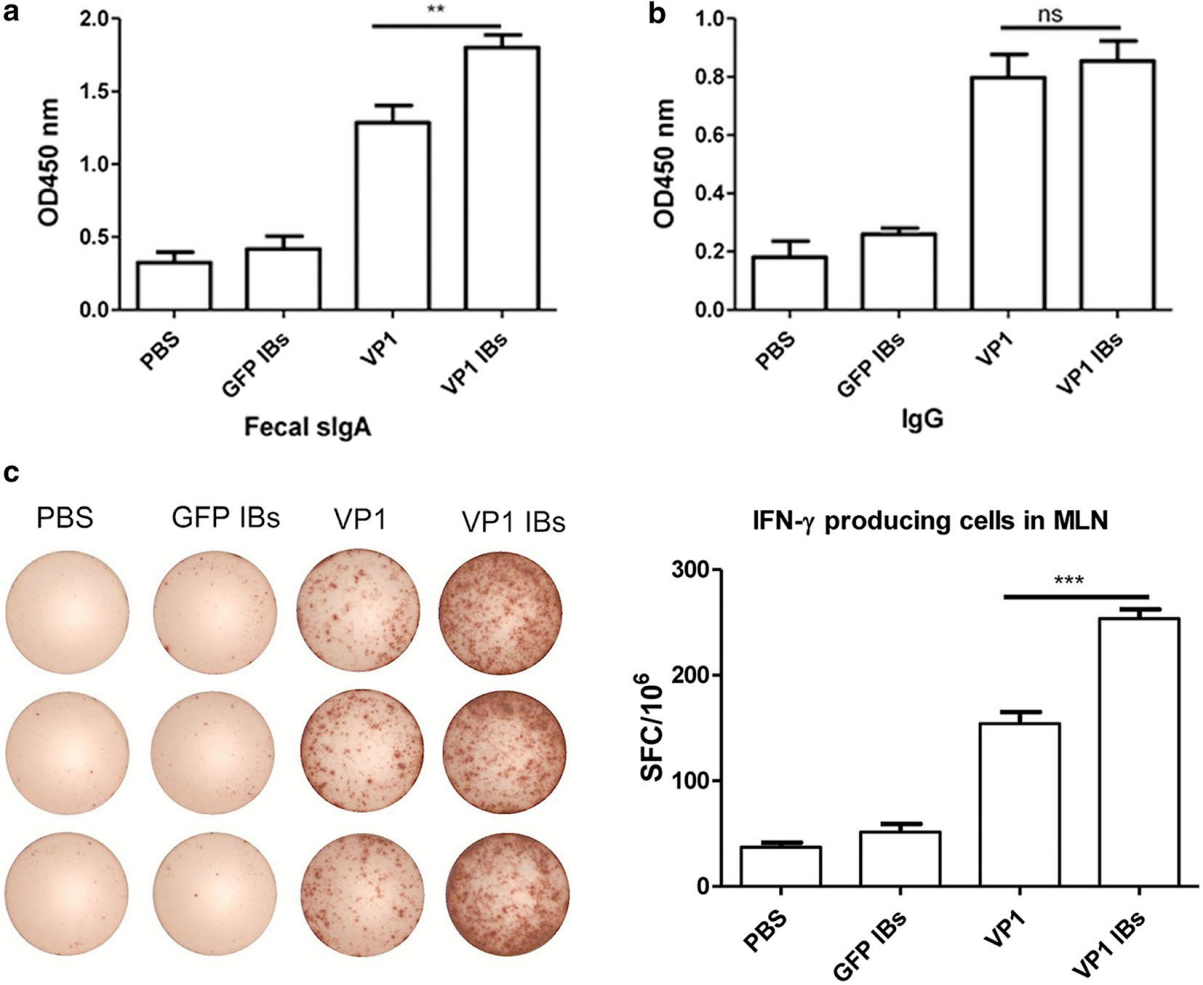
CVB3-specific immune responses induced by oral administration of VP1 IBs. **a** CVB3-specific fecal IgA level. **b** CVB3-specific serum IgG level. **c** Frequency of IFN-ɣ producing cells in MLN. **P < 0.01; ***P < 0.001

Induction of efficient mucosal immunity is extremely important, which can prevent enteric pathogen infection and invasion through mucosal surface [47]. Therefore, we oral administrated mice with VP1 IBs and evaluated the CVB3-specific fecal secretory IgA (sIgA). As shown in Fig. 5a, immunization with VP1 IBs exhibited a high CVB3-specific fecal sIgA antibody response compared with that of mice immunized with VP1 protein. To evaluate the ability of VP1 IBs to induce CVB3-specific mucosal T cell immunity, IFN-ɣ-producing T cell frequency in MLN were evaluated. As shown in Fig. 5c, VP1 IBs elicited much more IFN-ɣ-producing T cell in the MLN, the frequencies reaching 253 SFC/10^6^ cells, substantially higher than that of VP1 protein (154 SFC/10^6^ cells). As to the serum IgG level, no significant difference was observed between the VP1 IBs and VP1 protein group (Fig. 5b). These data indicated that oral immunization with VP1 IBs could generate efficient CVB3-specific mucosal immune response but failed to significantly enhance systemic immune response.

### Oral immunization with VP1 IBs significantly enhanced the immune-protection against CVB3-induced myocarditis

**Fig. 6:**
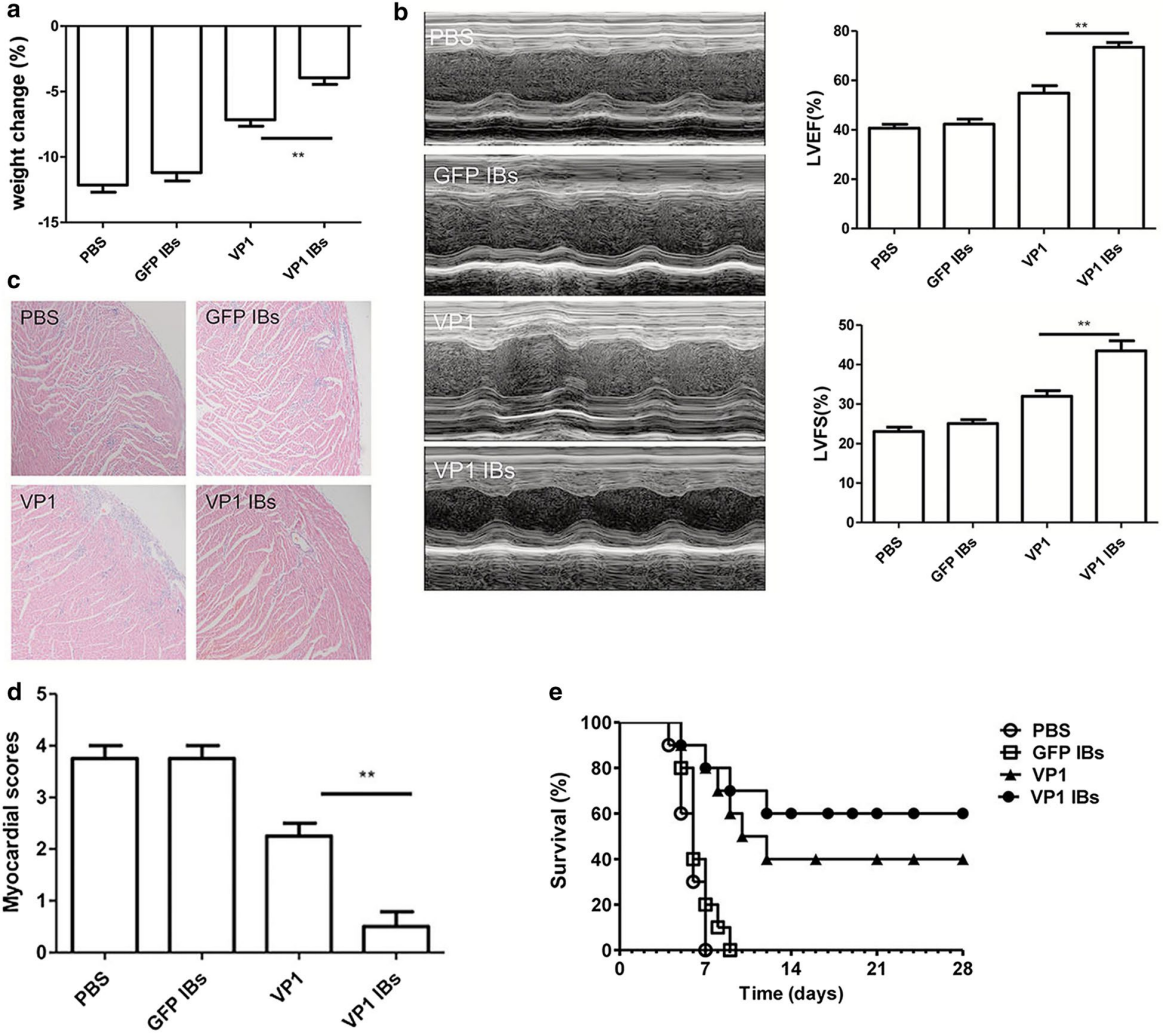
Enhanced resistance to CVB3-induced acute myocarditis by oral immunization with VP1 IBs. Myocarditis severity was determined 7 days post-viral infection. **a** Body weight loss. **b** Cardiac function was detected by echocardiography using a 2-dimensional guided M-mode ultrasound system for each group. **c** Representative myocardial histopathological observation. **d** Myocardial histopathological scores. **e** The survival rate of mice was observed for 28 days following a lethal dose of CVB3 infection. **P < 0.01

To detect the immune-protection of vaccine candidates, 2 weeks after the last immunization, mice were challenge with CVB3 to induce acute myocarditis. Seven days post-viral infection, the parameters reflecting the severity of viral myocarditis were evaluated including body weight loss, ventricular systolic function as well as myocardial histological observation. As shown in Fig. 6a, most slightly changed body weight loss was observed in VP1 IBs immunized group. In vivo ventricular systolic function was measured by fractional shortening (FS) and ejection fraction (EF) using an echocardiography assay (Fig. 6b). Compared with VP1 protein immunized mice, the left ventricular ejection fraction (LVEF) in VP1 IBs immunized mice was ~ 18.6% higher. When left ventricular fractional shortening (LVFS) was calculated, it was ~ 11.5% higher in VP1 IBs immunized mice compared with that in the VP1 protein immunized mice. Compared with the PBS or GFP IBs group, histological analysis of HE-stained heart sections showed moderate myocardial inflammatory infiltration and necrosis in the VP1 protein immunized mice, while tiny inflammation was seen in VP1 IBs immunized mice (Fig. 6c). The lower pathological score was also observed in VP1 IBs immunization group compared to VP1 protein immunization group (Fig. 6d).

To further evaluate the immune protection effect of the vaccine, mice were challenged with a lethal dose of CVB3 2 weeks after the final immunization. All mice in mock group died within 7 days, while 40% (4/10) of VP1 immunized mice and 60% (6/10) of VP1 IBs immunized mice survived to day 28 post infection (Fig. 6e). These data indicated that VP1 IBs exhibited enhanced immune protection against CVB3-induced myocarditis.

### In vivo uptake of VP1 IBs by murine Peyer’s patches (PPs)

**Fig. 7:**
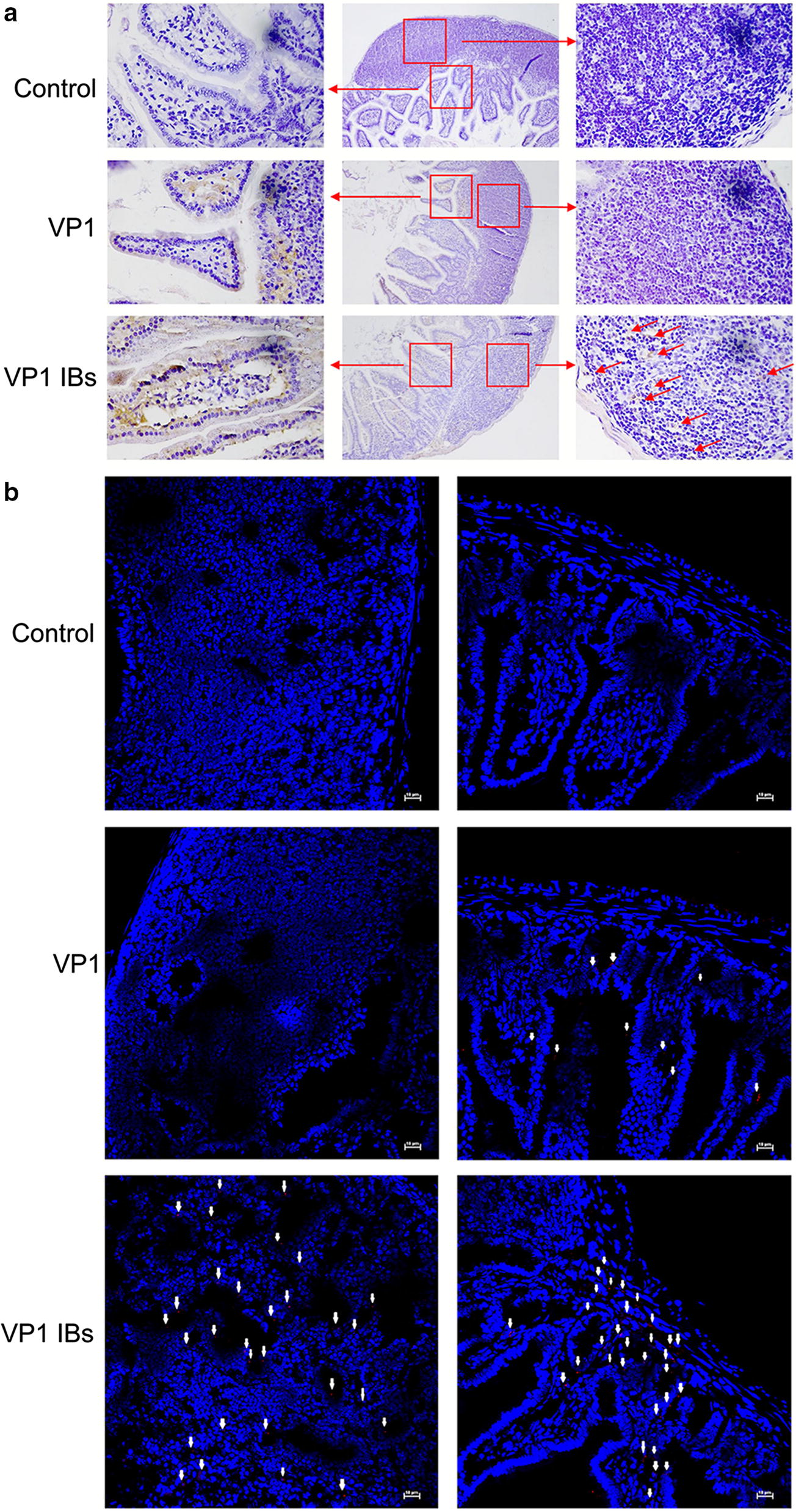
In vivo uptake of VP1 IBs by mouse PPs. Mice were oral administrated with 50 μg of VP1 IBs or VP1 protein, 12 h later the PPs were collected and subjected to immunohistochemistry (**a**) or immunofluorescence (**b**) analysis to detect protein uptake in vivo. The control group was carried out with the same steps, but the anti-VP1 antibody was replaced by PBS. Arrows indicate the VP1 protein or VP1 IBs

To uncover the mechanisms underlying the enhanced immune-protection against CVB3-induced myocarditis elicited by the VP1 IBs vaccine, we examined the uptake and distribution of antigens in the PPs through immunohistochemistry and immunofluorescence. PPs has been considered the major site of particle uptake due to the presence of microfold (M) cells. As shown in Fig. 7a, after oral administration, the amount of VP1 IB nanoparticles were much great than that of VP1 protein in the PPs. Meanwhile, the VP1 IBs were observed both in the subepithelial dome and the epithelial cell of PPs, the VP1 protein was only internalized by the epithelial cell overlying PPs. Furthermore, the efficient uptake of VP1 IBs by PPs was confirmed by the immunofluorescence, in which the fluorescence from VP1 IBs was observed in the PPs at much great levels than that of VP1 protein (Fig. 7b). These data demonstrated that the VP1 IBs as vehicles for oral vaccine could well protect the antigen from degradation in the gastrointestinal (GI) tract to reach the subepithelial dome of PPs to elicit a mucosal immune response.

### Oral immunization with VP1 IBs promotes dendritic cell (DC) maturation

**Fig. 8:**
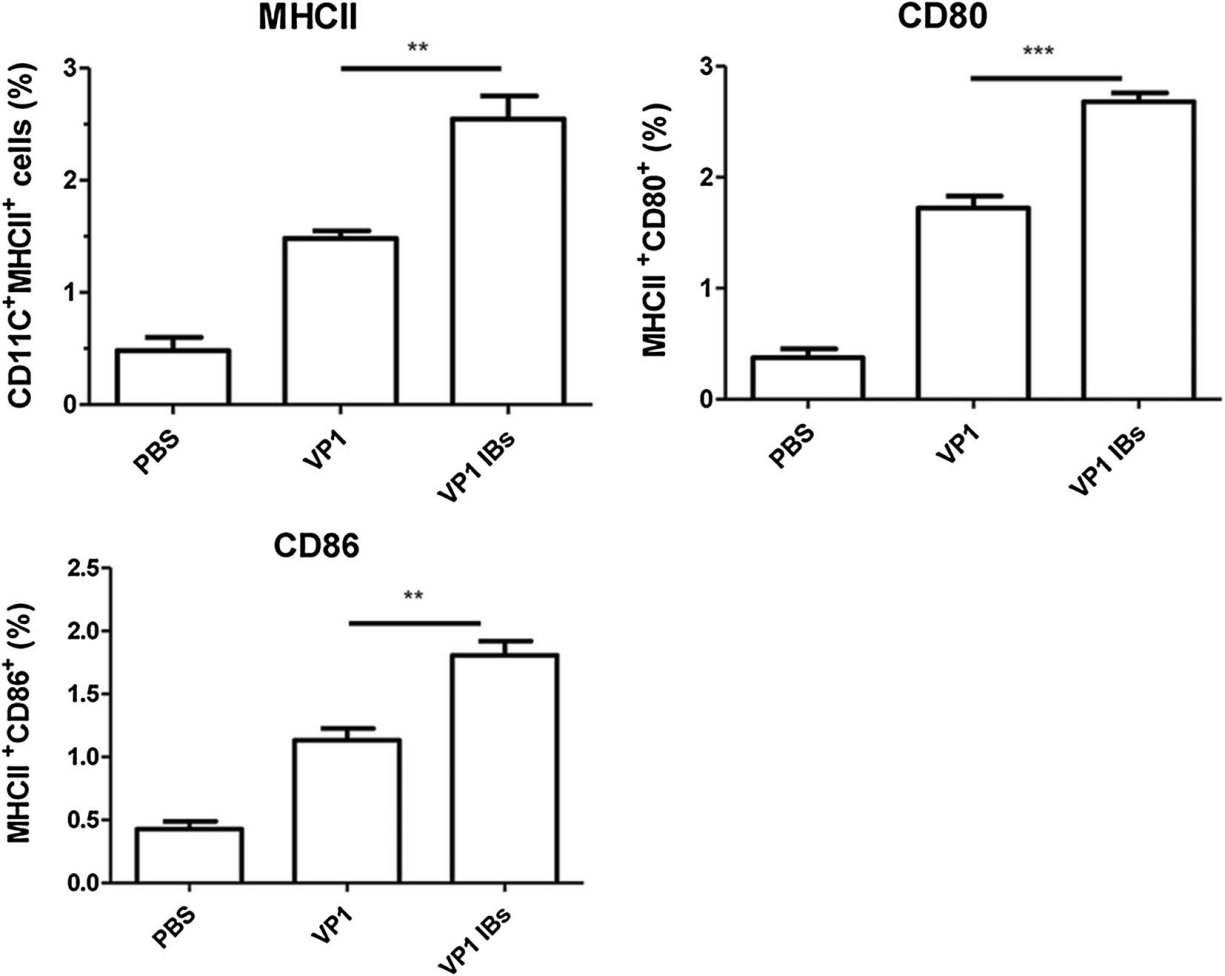
VP1 IBs immunization promoted MLN DCs maturation. Two weeks after last immunization, MLN cells were collected and stained with DC maturation markers CD80, CD86 and MHCII to determine by flow cytometry. **P < 0.01, ***P < 0.001

Since we observed that the vaccines differentially affected antigen transport to the PPs, we next wondered how they each affected the maturation of DCs in the MLN. As shown in Fig. 8, the much higher expression levels of costimulatory molecules CD80 and CD86 as well as the expression of MHCII were evidenced in VP1 IBs group compared to VP1 protein group, indicated that VP1 IBs immunization could induce effective mucosal DC maturation and lead to the enhancement of antigen-specific mucosal immune responses.

## Discussion

Mucosal vaccination, especially through oral administration route, is highly desired for infectious disease which is caused by pathogens infection through the mucosal surface. With the development of protein-based mucosal vaccine, the suitable vaccine delivery system is imperative for the successful protection of protein antigens to overcome their degradation by digestive enzymes present in the GI-tract and induction of sufficient mucosal immune responses. Among various potential delivery systems, IBs isolated from *E. coli* cells are excellent candidates to be used as protein-based delivery system by the oral route, as they are stable under gastrointestinal pH conditions in vivo and have the suitable nanostructure to be taken up by the gut-associated lymphoid tissue (i.e., Peyer’s patches) [18, 49]. CVB3 as a leading causative agent of viral myocarditis initiates their infections at the mucosal surface of the gastrointestinal. Therefore, orally delivered protein vaccine is high desired for CVB3-induced myocarditis. Taken together, we propose the VP1 protein as inclusion bodies isolated from recombinant *E. coli* as a candidate oral vaccine for CVB3-induced myocarditis. However, VP1 protein is difficult to express in *E. coli* without fusion tag (described in Additional file 1: Fig S1). In this work, we explored a tag-free VP1 IB nanoparticles production protocol though a truncated *Ssp DnaX* mini-intein spontaneous C-cleavage in vivo and also exploited the VP1 IBs as an oral protein nanoparticle vaccine to protect mice against CVB3-induced myocarditis. During the protein expression in *E. coli*, the fusion precursor protein (Trx-Ic-VP1) divides into two parts through intein spontaneous C-cleavage, the fusion partner (Trx-Ic) as soluble protein in the supernatants and the antigen (VP1) as inclusion bodies in the pellets, which facilitates to be separated. In general, as a self-cleaving fusion tag, intein lack the capacity for improving target protein expression. In order to get efficient protein expression, we fused Trx to the N terminus of I_C138_ gene sequence. Meanwhile, effective cleavage depends on a correct folding intein and the fusion precursor protein needs to be expressed in a soluble form. To overcome these problems, low temperature cultivation and low IPTG concentration are widely performed and these have proved to be effective strategies in our study to successfully produce tag-free VP1 IBs [50, 51]. On the contrary, at high temperature (37 °C) or high IPTG concentration (0.5 mM or 1 mM) the fusion protein mainly expressed as precursor proteins (described in Additional file 1: Fig S2).

The uptake of nanoparticles by PPs is an important step for determining the efficacy of oral immunization and particle size is one of the most important factors for the uptake of nanoparticles into the PPs subepithelial dome. It is now generally accepted that particles < 1 um can be efficiently taken up by PPs [52, 53]. Since IB particles have a particulate characteristics, such as nanoscale size (range from 50 to 1000 nm) and much less vulnerable to degradation by enzymes and acids, that IB particle antigen should be taken up more efficiently than soluble protein by PPs [54, 55]. This may explain the efficacy uptake of VP1 IBs in the subepithelial dome of PPs by oral administration, while VP1 soluble antigen is only observed in PPs follicle associated epithelium (FAE). On account of its strong uptake by the PPs subepithelial dome, mice oral immunization with VP1 IBs without adjuvant induced fecal IgA response. This is of special importance science sIgA immune response is of special interest in the development of vaccines against pathogenic microorganisms invading mucosal sites [56]. The usefulness of IBs in oral immunization was also demonstrated in other studies, which also showed the efficient uptake by cells of the intestinal mucosa and induced efficient mucosal response [49, 57]. As a result of typical immune response, immunization with VP1 IBs are able to efficiently protect mice from CVB3-induce myocarditis. Moreover, we have obtained higher survival percentages when challenged with a lethal dose of CVB3. The higher efficiency mucosal immune response induction by VP1 IBs without any adjuvant compared to that of VP1 soluble protein could be due to the differential presence of co-purified bacterial components (LPS, lipid and nucleic acids) besides the particulate characteristics of IBs. Such compositional complexity combined in a single particle provides a different form of immunostimulant to enhance the immune protection. Although as naturally compositional heterogeneity, IBs do not show any detectable signs of local or systemic toxicity in mice by oral delivery of IBs [29]. In repeated administration, VP1 IBs were well tolerated by all animals and no histologically damage in the intestine was observed in IB-fed animals compared to control animals. Vaccination by the oral route can induce both mucosal and systemic immune responses. In our experiment oral immunization with VP1 IBs could obviously increase CVB3-specific mucosal but not systemic immune responses compared with VP1 protein vaccine. This may due to the low dose of inclusion bodies used for immunization by oral route. Previously study have shown that oral immunization IBs with a single 100 μg does could induce a more robust plasma antibody response, while two 50 μg doses of antigen in inclusion bodies developed significant levels of IgA antibodies and could establish long-term mucosal memory [57]. It has been well-accepted that mucosal immune response elicited by oral vaccine is more important for controlling mucosally invaded pathogen infection compared with systemic immunity. Therefore, double immunization with 50 μg of VP1 IBs per dose was chosen to perform in our study.

## Conclusions

In conclusion, the exploitation of tag-free IBs as carriers for oral particle-based subunit vaccine offer many advantages. First, these bacterial IBs are produced in recombinant bacteria which are relatively easy to manipulate and have high capacity to express foreign genes and show rapid growth. Second, they have been shown to be highly stable over time to resist different harsh conditions as well as long-term storage. Third, many physicochemical parameters of the material are fully controllable by both genetic and process engineering. Forth, and more importantly, they do not show any sign of toxicity despite their compositional heterogeneity and naturally cell penetrability and sustained release of active proteins to the cytosol of the uptaking cells. Collectively, all these aspects make IBs a highly versatile option with excellent potential to be used as a nano-sized protein vaccine against a wide range of infectious diseases. In this study, we only employed CVB3 VP1 as a model protein to test the spontaneous C-cleavage of I_C138_ of *Ssp DnaX* mini-intein to production tag-free IBs in vivo. In our previous study, we have shown that this system could undergo spontaneous C-cleavage nearly completely when fused with Trx or GFP proteins [45], although Trx and GFP expressed as soluble forms, which also indicated that this system may have universal applicability to a variety of proteins that produced tag-free inclusion body particles.

## Methods

### Plasmids construction

As illustrated in Fig. 1, plasmid pMSX-I_C138_-VP1 was derived from plasmid pMSX-I_C138_ [45] by replacing the thioredoxin (Trx) coding sequence on an *Age*I–*PstI* fragment with a VP1 coding sequence that was isolated as an *AgeI*–*PstI* fragment from the previously reported pcDNA3.1-VP1 plasmid [58]. Plasmid pMSX-Trx-I_C138_-VP1 was derived from plasmid pMSX-I_C138_-VP1 by adding a *NdeI*–Trx–*NdeI* fragment to the N terminus of I_C138_ gene sequence. As the first residue of native C-extein (Cys for *Ssp DnaX* intein) is essential for intein splicing or cleavage, we added the amino acid Cys to the N terminal of VP1 protein for the plasmid pMSX-Trx-I_C138_-VP1. These recombinant plasmids were all verified through DNA sequencing. *E. coli* DH5α was employed in all the cloning work.

### Tag-free VP1 IBs expression and purification

For protein expression, recombinant plasmid pMSX-Trx-I_C138_-VP1 was introduced into *E. coli* BL21 (DE3). Cells were grown in LB medium containing ampicillin to an OD600 of ~ 0.6, and protein expression was induced with 0.25 mM IPTG at room temperature for 16 h, then harvested and lysed in reducing SDS sample buffer. Western blotting analysis was carried out using 6× His tag antibody (anti-H, Sigma) and VP1 antibody (anti-VP1, Dako).

For IBs purification, the bacterial cultures (500 ml) were harvested by centrifugation at 5000*g* at 4 °C for 30 min and resuspended in 30 ml of lysis buffer (50 mM Tris–HCl pH 8.0, 100 mM NaCl and 1 mM EDTA) and frozen at − 80 °C. After thawing, the mixture was sonicated on ice (Branson Sonifier 450, USA). The resulting cell lysate was centrifuged at 12,000*g* for 20 min. The supernatant (S) and the insoluble pellets (P) were retained for analysis by SDS–PAGE, respectively. In order to obtain pure and cell free VP1 IBs, the insoluble pellets were used for purification as previously described [59]. Briefly, lysozyme (1 μg/ml) and phenylmethanesulfonyl fluoride (PMSF, 0.4 mM) were added to the pellets and incubated at 37 °C for 2 h. Then, the pellets were washed extensively for four times with 30 ml of washing buffer, followed by addition of DNase (1 μg/ml) and incubated at 37 °C for 1 h. Next, freeze/thaw cycles were repeated until no viable bacteria were detected. For this, 100 μl of the culture was seeded in LB plates without antibiotic and cultivated overnight at 37 °C. Finally, the IBs were washed with PBS to remove contaminating detergent and centrifuged at 12,000*g* for 20 min and the purity IBs were stored at − 80 °C until use. IBs were semi-quantified by Western Blot densitometry (Image J) using anti-VP1 antibody (Dako). Note that GFP IBs were produced as the same procedure and as control IB nanoparticles with irrelevant biological activity regarding immunization.

### VP1 IBs characterization

Purified VP1 IBs were characterized by confocal microscopy and scanning electron microscopy (SEM). To visualize VP1 IBs by confocal microscopy, FITC (Sigma) was conjugated at a molar ration 1:2 (protein/dye) following manuscript instructions. Samples of FITC-labeled VP1 IBs (FITC-VP1 IBs) were placed on a glass slide, fixed with a slide cover and observed with a Nikon A1 (Japan) confocal fluorescence microscope. For electron microscopy scanning, purified VP1 IBs were thoroughly washed in pure water and resuspended in ethanol. Samples were prepared on a gold-coated silicon and observed under a Hitachi scanning electron microscope (Japan, S-4700). IBs nanoparticle size distribution was obtained by measuring the diameter of 150 particles from SEM micrographs using the free software Image J.

### Mice and virus

All animal studies were performed according to the Guide for the Care and Use of Medical Laboratory Animals (Ministry of Health, China, 1998) and with the ethical approval the guide of Soochow University. Male BALB/c mice were purchased from the Experimental Animal Center of Chinese Academy of Science (Shanghai, China) and used at 6–8 weeks of age. CVB3 virus (Nancy strain) was prepared by passage through HeLa cells.

### VP1 IBs uptake by bone marrow-derived dendritic cells (BMDCs)

BMDCs were generated as previously described [60]. Briefly, the femurs and tibias were rinsed in 70% ethanol, epiphyses removed, and the marrow flushed. Red blood cell (RBC)-depleted BALB/c bone marrow cells were plated at 2 × 10^5^ cells/ml (10 ml total) on sterile bacteriological Petri dishes (Fisher) in complete RPMI media supplemented with 20 ng/ml murine recombinant GM–CSF and 20 ng/ml murine recombinant IL-4 (DC media). Cells were maintained at 37 °C and 5% CO_2_, and 10 ml fresh DC media was added on day 3. On day 6, 50% of the media was replaced, and the non-adherent cells were pelleted and added back to the plates. Loosely and non-adherent cells were collected and used as inactivated BMDCs on day 8.

The uptake of VP1 IBs by BMDCs was analyzed by confocal microscopy. BMDCs were plated at 5 × 10^5^ cells/well in 24-well plates and allowed to settle overnight. The FITC-VP1 IBs (10 μg) were added and incubated with cells for 12 h. After incubation, the medium was discarded, and the cells were washed with PBS for three times. Then, the nuclei and membranes were labeled with Hoechst 33342 (20 μg/ml) and CellMask™ Deep Red (2.5 μg/ml) (both from Molecular Probes) for 10 min at room temperature, and washed twice prior to confocal detection. The samples were examined with a Nikon A1 (Japan) confocal fluorescence microscope. 3D reconstructions were done as described [29].

### T cell proliferation assay in vitro

Briefly, for T cell proliferation assays, the spleens were crushed through a 70-mm cell strainer in ice cold PBS and centrifuged at 300*g* for 5 min. RBCs were depleted with ACK lysing buffer. T cells were suspended at 3 × 10^6^ cells/mL in PBS containing 5 uM of the intracellular dye CFSE and incubated at room temperature for 20 min. The reaction was quenched by 10-fold dilution in RPMI containing 10% FBS and cells were washed twice with PBS. Freshly prepared CFSE-labeled T cells were added to the antigen-stimulated BMDCs at 3 × 10^5^ cells/well (10:1 T cells:DCs) and cultured at 37 °C for 72 h. Antigen-stimulated BMDCs (3 × 10^4^ cells/well) were prepared by incubating with VP1 IBs for 4 h and then washed with PBS to remove excess antigen. Cells were harvested and analyzed by flow cytometry using a BD FACS Canto™II instrument for CFSE dilution of T cells.

### Cytotoxicity assay

HeLa cells were routinely cultured in DMEM 10% FBS (Gibco, UK) and 2 mM l-glutamine (Gibco, UK) at 5% CO_2_ at 37 °C in a humidified incubator. 3 × 10^4^ HeLa cells were seeded per well in 96 well plates. After 24 h of incubation, 0, 5, 10 and 20 μg of purified VP1 IBs were added and cell cultures further incubated for 24 h. Cytotoxicity was then measured in the culture with the Cytotoxicity Detection Kit^PLUS^ (LDH, Roche). All cell samples were tested in duplicate.

### Tolerance of mice to oral VP1 IBs administration

Three groups of animals were used to ascertain whether repeated dosing of VP1 IBs would be well tolerated. One group was treated by oral administration for 4 days with 200 μl of PBS, two other groups were equally treated with 50 μg or 100 μg VP1 IBs suspended in 200 μl PBS. The animal body weight and food intake, behavior and aspect were monitored daily. Four days later, mice were euthanized via decapitation. The abdomen was opened and the intestines were collected. The tissues were fixed in 10% phosphate-buffered formalin, paraffin embedded, sectioned and stained with H&E.

### Oral administration of VP1 IBs

Mice were orally immunized with vaccines (VP1 protein, VP1 IBs or GFP IBs) containing 50 μg purified proteins suspended in 200 μl PBS for twice with a 2-week interval, with GFP IBs as a control of an IB without a relevant immune role, and with VP1 protein as a soluble protein vaccine. Control mice were mock-immunized with 200 μl PBS according to the same schedule. Experimental groups consisted of a minimum of six mice, and experiments were repeated at least twice. Serum and fecal samples were collected 2 weeks following the final immunization and used for evaluation of antibody. Fresh fecal pellets were dissolved in PBS containing BSA and protease inhibitors (1 mg/mL aprotinin, 1 mM PMSF) at final concentration of 100 mg/ml. After centrifugation at 15,000*g* for 10 min, supernatants were collected and stored at − 80 °C.

### ELISA measurement of CVB3-specific antibody

Plasma and fecal extracts were assayed for the presence of antibodies against VP1 antigen by an ELISA method. Plasma samples were tested at a dilution of 1:10 and HRP-conjugated goat anti-mouse IgG diluted 1:1000 was used as a secondary antibody. Fecal extracts were analyzed undiluted and secondary labeling was done using HRP-linked goat anti-mouse IgA at 1:1000 dilution. Plates were coated with 10 μg/ml VP1_237–249_ peptide (GL Biochem Corp, Shanghai) at 4 °C overnight. After blocking with 5% non-fat milk in PBS, serum or fecal samples were added and incubated at 37 °C for 2 h. After washing the plates three times, HRP-conjugated goat anti-mouse IgG or IgA (Southern-Biotech) was added, followed by TMB substrate addition. After incubation for 30 min at room temperature, the reaction was stopped by the addition of 0.5 M sulfuric acid and the absorbance was quantified at 450 nm by a microplated reader (Bio-Lab).

### IFN-γ ELISPOT assay

Two weeks after the final immunization, mesenteric lymph node (MLN) cells from immunized mice were isolated for assessing of IFN-γ producing cell frequency using IFN-γ ELISPOT kit (BD Pharmingen). Briefly, cells were plated at 1 × 10^6^ cells/well and stimulated with VP1_237-249_ protein (10 μg/ml) for 48 h at 37 °C with 5% CO_2_. After sequential incubation with biotinylated detection antibody, streptavidin-HRP and AP-colorimetric substrate, color was developed and spot-forming cells were enumerated with an ImmunoSpot Series 3 Analyzer (Cellular Technology).

### CVB3 infection and myocarditis evaluation

Two weeks after the final immunization, the immunized mice were infected by intraperitoneally with 3× 50% lethal dose (LD50) CVB3. After 7 days later, cardiac function was assessed as previously described [58]. Then, hearts were collected, sectioned and stained with hema-toxylin and eosin (HE). The Histopathological changes were compared quantitatively by calculating the histopathological scores. For survival rate, ten mice each group were administrated with a lethal dose of CVB3 (5LD50) and survival was monitored until day 28.

### Immunofluorescence and Immunohistochemistry staining of VP1 IBs in the Peyer’s patches (PPs) of mice

Mice were oral administrated with 50 μg VP1 IBs suspended in 200 μl PBS. After 12 h, the PPs were dissected and fixed in 10% phosphate-buffered formalin, then paraffin embedded. Sections of 0.6 um were obtained and stained with mouse anti-VP1 antibody (Dako) followed by cy3-labled goat anti-mouse IgG secondary antibody (Abcam) or by biotinylated goat anti-mouse IgG secondary antibody (Abcam). The negative control group was carried out with the same steps, but the anti-VP1 antibody was replaced by PBS.

### The maturation of DCs detection by flow cytometry

The maturation of the mesenteric lymph modes (MLN) DC was examined in the immunized mice using flow cytometry. Two weeks after final immunization, MLN cells were isolated and stained with a DC myeloid marker, FITC conjugated anti-mouse CD11c (Biolegend) and one of the following maturation markers (Pecy7 conjugated anti-mouse MHCII, PerCP conjugated anti-mouse CD80, APC conjugated anti-mouse CD86; Biolegend). The percentages of CD11c^+^MHCII^+^, CD11c^+^CD80^+^ and CD11c^+^CD86^+^ cells were determined by flow cytometry using a BD FACS Canto™II instrument. All data were analyzed using FlowJo software version 7.6.

### Statistical analysis

Data were presented as mean ± SEM. Statistical analysis was performed with ANOVA followed by Tukey’s post hoc test. Survival rates were analyzed by Kapla-Meier test using GraphPad Prism version 5.01 (GraphPad Software Inc.). P < 0.05 was considered statistically significant.

## Additional file

**Additional file 1.** Supplementary material.

## Abbreviations

IBs: inclusion bodies
Trx: thioredoxin
CVB3: Coxsackievirus B3
VP1: CVB3 capsid protein-1
BMDCs: bone marrow-derived dendritic cells
PPs: Peyer’s patches
MLN: mesenteric lymph node
APCs: antigen presenting cells
SEM: scanning electron microscopy
