# Supplementary material for: Spontaneous C-cleavage of a truncated intein as fusion tag to produce tag-free VP1 inclusion body nanoparticle vaccine against CVB3-induced viral myocarditis by the oral route
Source: Microb Cell Fact. 2019 Apr 4;18:66. doi: 10.1186/s12934-019-1115-z (PMC6449988; doi:10.1186/s12934-019-1115-z)
Supplement: Supplementary file 1 — Additional file 1. Supplementary material. [file 12934_2019_1115_MOESM1_ESM.docx]

**Additional information**

**Spontaneous C-cleavage of a truncated intein as fusion tag to produce tag-free VP1 inclusion body nanoparticle vaccine against CVB3-induced viral myocarditis by the oral route**

Xingmei Qi, Qian Lu, Jing ping Hu and Sidong Xiong^*^

Jiangsu Key Laboratory of Infection and Immunity, Institutes of Biology and Medical Sciences, Soochow University, Suzhou, Jiangsu 215123, China

**Corresponding author:** Sidong Xiong

Email addresses:

Sidong Xiong: sdxiongfd@126.com

Xingmei Qi: xmqi@suda.edu.cn

Qian Lu: 1169525212@qq.com

Jingping Hu:jingpinghu@suda.edu.cn

**Figure S1**

**The VP1 protein expression without fusion tag in *E.coli***

The recombinant plasmids pMSX-VP1 and pMSX-Trx-I_C138_-VP1 were introduced into *E.coli* BL21(DE3) cells and induced by IPTG to express the corresponding recombinant protein, VP1 and Trx-I_C138_-VP1, respectively. The total cellular proteins were analyzed by SDS–PAGE followed by staining. As shown in Fig. S1, compared to the pMSX-Trx-I_C138_-VP1 plasmid construction, there only trace amount of VP1 protein expression from the construction of pMSX-VP1.The results indicated that the Trx-intein fusion was helpful in increasing expression over the VP1 alone.

***
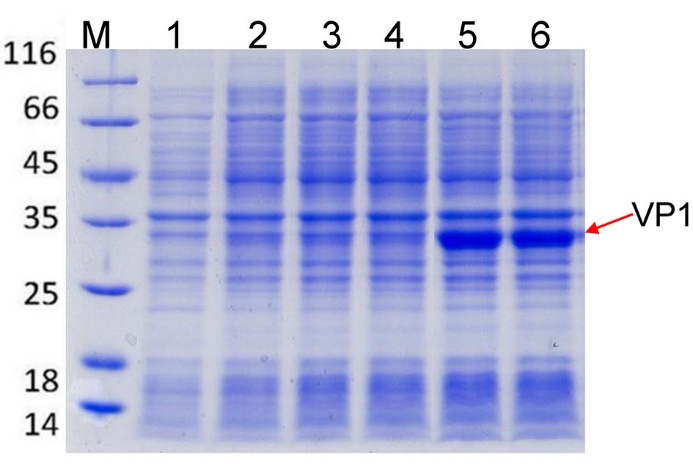
***

Figure S1. SDS–PAGE analysis of the protein expression from plasmids pMSX-VP1 and pMSX-Trx-I_C138_-VP1 in *E.coli*. Lane M: protein size markers, with their sizes shown in kDa. Lanes 1, un-induced protein expression. Lane 2: induced plasmid pMSX-VP1 protein expression at 25°C and IPTG 0.5mM for 16 h. Lane 3: induced plasmid pMSX-VP1 protein expression at 25°C and IPTG 1 mM for 16 h. Lane 4: induced plasmid pMSX-VP1 protein expression at 37°C and IPTG 1 mM for 4 h. Lane 5: induced plasmid pMSX-Trx-I_C138_-VP1 protein expression at 25°C and IPTG 0.25 mM for 16 h. Lane 6: induced plasmid pMSX-Trx-I_C138_-VP1 protein expression at 25°C and IPTG 0.5 mM for 16 h.

**Figure S2**

**The fusion protein expression and C-cleavage assay in *E.coli*.**

As effective cleavage depends on a functional intein, the intein needs to be expressed in a soluble, correctly folded form. We performed different conditions to induce the fusion protein expression and tested the intein C-cleavage efficiency. Spontaneous intein C-cleavage would convert the precursor protein Trx-I_C138_-VP1 (60 KDa) into Trx-I_C138_ (28 KDa) and VP1 (32 KDa). As shown in Fig. S2, compared to the un-induction protein (U), additional protein bands were identiﬁed after induction (I) by their apparent sizes in SDS–PAGE. From the results we could see that the protein expressed mainly as precursor protein at 37°C and IPTG 1mM, and about 50% precursor protein was converted to cleavage products (Trx-I_C138_ and VP1) at 25°C and IPTG 1mM, and about 90% precursor protein was converted to cleavage products at 25°C and IPTG 0.5 mM, and about 100% precursor protein was converted to cleavage products at 25°C and IPTG 0.25 mM form their apparent size.


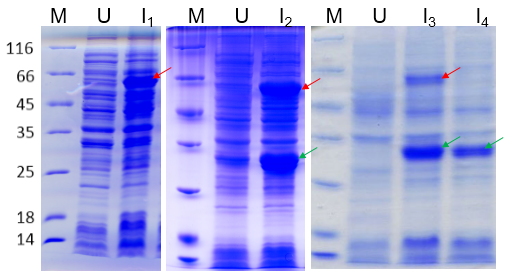


Figure S2. SDS–PAGE analysis of the C-cleavage of recombinant protein Trx-Ic_138_-VP1 in *E.coli*. Lane M: protein size markers, with their sizes shown in kDa. Lanes U and I: total cellular proteins of *E. coli* before and after IPTG-induced expression the Trx-Ic_138_-VP1 protein, respectively. I_1,_ induced protein expression at 37°C and IPTG 1mM; I_2,_ induced protein expression at 25°C and IPTG 1mM; I_3,_ induced protein expression at 25°C and IPTG 0.5 mM; I_4,_ induced protein expression at 25°C and IPTG 0.25 mM. The red arrow indicated the precursor protein and the green arrow indicated the cleavage product VP1 IB protein.
